# Designing an Electronic Patient Management System for Multiple Sclerosis: Building a Next Generation Multiple Sclerosis Documentation System

**DOI:** 10.2196/ijmr.4549

**Published:** 2016-01-08

**Authors:** Raimar Kern, Rocco Haase, Judith Christina Eisele, Katja Thomas, Tjalf Ziemssen

**Affiliations:** ^1^Multiple Sclerosis Center Dresden, Center of Clinical NeuroscienceDepartment of NeurologyUniversity of Technology Dresden, GermanyDresdenGermany

**Keywords:** health information technology, computers, Internet, multiple sclerosis, eHealth, disease management

## Abstract

**Background:**

Technologies like electronic health records or telemedicine devices support the rapid mediation of health information and clinical data independent of time and location between patients and their physicians as well as among health care professionals. Today, every part of the treatment process from diagnosis, treatment selection, and application to patient education and long-term care may be enhanced by a quality-assured implementation of health information technology (HIT) that also takes data security standards and concerns into account. In order to increase the level of effectively realized benefits of eHealth services, a user-driven needs assessment should ensure the inclusion of health care professional perspectives into the process of technology development as we did in the development process of the Multiple Sclerosis Documentation System 3D. After analyzing the use of information technology by patients suffering from multiple sclerosis, we focused on the needs of neurological health care professionals and their handling of health information technology.

**Objective:**

Therefore, we researched the status quo of eHealth adoption in neurological practices and clinics as well as health care professional opinions about potential benefits and requirements of eHealth services in the field of multiple sclerosis.

**Methods:**

We conducted a paper-and-pencil–based mail survey in 2013 by sending our questionnaire to 600 randomly chosen neurological practices in Germany. The questionnaire consisted of 24 items covering characteristics of participating neurological practices (4 items), the current use of network technology and the Internet in such neurological practices (5 items), physicians’ attitudes toward the general and MS-related usefulness of eHealth systems (8 items) and toward the clinical documentation via electronic health records (4 items), and physicians’ knowledge about the Multiple Sclerosis Documentation System (3 items).

**Results:**

From 600 mailed surveys, 74 completed surveys were returned. As much as 9 of the 10 practices were already connected to the Internet (67/74), but only 49% preferred a permanent access. The most common type of HIT infrastructure was a complete practice network with several access points. Considering data sharing with research registers, 43% opted for an online interface, whereas 58% decided on an offline method of data transmission. eHealth services were perceived as generally useful for physicians and nurses in neurological practices with highest capabilities for improvements in clinical documentation, data acquisition, diagnosis of specific MS symptoms, physician-patient communication, and patient education. Practices specialized in MS in comparison with other neurological practices presented an increased interest in online documentation. Among the participating centers, 91% welcomed the opportunity of a specific clinical documentation for MS and 87% showed great interest in an extended and more interconnected electronic documentation of MS patients. Clinical parameters (59/74) were most important in documentation, followed by symptomatic parameters like measures of fatigue or depression (53/74) and quality of life (47/74).

**Conclusions:**

Physicians and nurses may significantly benefit from an electronically assisted documentation and patient management. Many aspects of patient documentation and education will be enhanced by eHealth services if the most informative measures are integrated in an easy-to-use and easily connectable approach. MS-specific eHealth services were highly appreciated, but the current level of adoption is still behind the level of interest in an extended and more interconnected electronic documentation of MS patients.

## Introduction

### Background

Numerous promising opportunities for patients and physicians are associated with an elaborate and concerted integration of health information technology (HIT) in everyday health care of clinics and practices [1-5]. HIT commonly comprises hardware devices and software applications supporting health-related information sharing, decision making, and health behavior. Technologies like electronic health records (EHRs) and telemedicine devices facilitate the rapid mediation of health information and clinical data independent of time and location between patients and their physicians as well as among health care professionals. Today, every part of the treatment process from diagnosis, treatment selection, and application through patient education and long-term care may be enhanced by a quality-assured implementation of HIT that also takes data security standards and concerns into account [6-9].

In addition to patients and healthcare professionals, further agents in the field of health management and their particular interests must be considered when designing and maintaining a comprehensive health-related electronic application. At the level of nationwide health care systems, eHealth technologies provide a substantial potential for cost control, cost savings, and rapid responses to public health emergencies [10-14]. Furthermore, researchers and industry representatives have been showing an increased interest in data liquidity being encouraged by the prospects of widely and securely available patient data; “big data” techniques may improve the cooperation and work flow between researchers and create innovation platforms for an exchange of ideas and, of course, real world health data [15-18].

In an ideal scenario of well-connected health professionals, the EHR serves as key source of health information for physicians, patients, and other users of the health care system infrastructure comprising multimodal information from heterogeneous domains and making them accessible according to the needs of all users and the connection standards of the research network [1,19-22].

Beyond the use of EHRs for documentation and information sharing on an individual and organizational level, the perspective of long-term care and management of chronic diseases extends the concept of complex health technologies by the dimension of time. As representative of such technologies, patient-centered electronic disease management systems have gained in importance over the last few years aiming to support individual care plans and physician-patient communication by evidence-based and standardized treatment guidelines [23].

Multiple sclerosis (MS) is one of the most frequent chronic neurological diseases showing first symptoms between the second and fourth decade of life [24,25]. Due to the long duration of the disease, its early onset, and the increase in therapeutic options, physicians need to establish individualized therapeutic approaches including long-term documentation and patient management over several years [26,27]. These characteristics demonstrate the need for a well-structured health information management and an opportunity to advance health care by innovative HIT. Consequently, the amount of eHealth services for MS has been growing over the past decade. MS patients have become used to information sharing and seeking via the Internet [28-31]. Several MS-specific electronic networks and databases have been established forwarding health information between patients and toward researchers [32-38]. Furthermore, there is a growing trend to analyze data generated from EHRs [39-41]. Standardized therapy documentation provides a solid foundation for data mining from EHRs as well as for disease management. Therefore, electronic large-scale documentation systems with standardized interfaces like the Multiple Sclerosis Documentation System 3D (MSDS^3D^, successor of the most widely used MS documentation system in Germany) constitute a promising way of aiding empirical medical research and translational health care [26,42-47].

Today, securing benefits of HIT at as many levels as possible and simultaneously precluding technology-immanent obstacles like the unfortunate exclusion of users (due to digital divide), data insecurity, and inefficient implementation (as a result of incompatibility between systems or double documentation) remain major tasks in the development of eHealth applications in general [2,48-51]. Considering this, an implementation of clinical pathways is a highly recommended strategy to realize both standardization and personalization in the treatment process [52-54]. Clinical pathways in HIT reliably comprehend data from diagnosis to treatment and enable controlling processes for quality and cost. In a multilevel approach like MSDS^3D^, health data is shared among physicians, nurses, and patients and integrated according to clinical pathways. Beyond that, data liquidity is increased by associated data management tools and the ability to connect with research registers.

Despite the given advantages and the rising number of EHR adopters, there is still a relevant number of nonadopters of EHR systems and professionals not using the full potential of modern HIT [51,55]. In order to avoid an isolation of physicians and patients not using recommended and widespread assisting HIT and to increase the level of effectively realized benefits, a user-driven needs assessment should ensure the inclusion of health care professional and patient perspectives into the process of HIT development. After analyzing the use of information technology by MS patients and their willingness to adopt it for therapy in a previous study, we focused on the needs of neurological health care professionals and their handling of HIT, especially of electronic patient management systems and EHRs [56-58].

### Objectives

With our exploratory survey among neurologists as an extension of our patient-oriented previous study, we researched the status quo of HIT adoption in neurological practices and clinics. In addition, we aimed to survey health care professionals’ opinions about potential benefits and important requirements of eHealth services in the field of MS treatment and documentation in order to enhance the user-driven development of an elaborate documentation and patient management system (MSDS^3D^). Furthermore, it had to be ascertained whether there are differences between universal neurological practices and MS-specific practices in terms of eHealth use and acceptance.

## Methods

### Participants

We conducted a paper-and-pencil–based mail survey at the Multiple Sclerosis Center Dresden (Dresden, Germany) in 2013 by sending our questionnaire together with a cover letter to 600 randomly chosen neurological practices in Germany. The cover letter provided information about the scope and the purpose of our survey (see Objectives). Physicians (as head of their neurological practice) were asked to anonymously fill in the 23 questions and return the survey via postal mail in an enclosed self-addressed prepaid envelope (1 questionnaire per practice). A short reminder with a download link to the survey file was also sent via postal mail 3 months after the initial mail. In doing so, we wanted to reach as many practitioners as possible without losing relevant opinions due to an unfavorable effect of technology-based preselection. Neurological practices specialized in MS and non–MS-specialized neurological practices as well as practices with small (less than 100 quarterly) and large (more than 200 quarterly) numbers of patients were included in the survey population.

### Questionnaire

The questionnaire was developed in a consensus meeting with a multiprofessional expert team consisting of physicians, psychologists, and computer scientists from the Multiple Sclerosis Centre Dresden as part of the University Hospital Carl Gustav Carus Dresden in a similar manner to the development of our previous questionnaire [56]. Items were selected with respect to the target audience (physicians and nurses) and the variety of tasks in the process of daily health care. With 23 items and subitems, we aimed to describe the participating neurological practices (4 items), the current use of network technology and the Internet in such neurological practices (5 items), physicians’ attitudes toward the general and MS-related usefulness of eHealth systems (8 items) and toward the clinical documentation via electronic health records (4 items), and physicians’ knowledge about the MSDS (3 items). Items were structured and combined single choice, multiple choice, and free text answers. For a translated English version, see Multimedia Appendix 1.

### Statistical Analysis

All statistical comparisons were two-tailed, and a *P* value of <.05 indicated statistical significance. We used SPSS version 22.0 (IBM Corp) for all statistical computations. Chi-square tests or Fisher’s exact tests (in case of expected cell counts lower than 5) were used for group comparisons of nominal data. Paired dichotomous data were analyzed using the McNemar’s test. For comparisons of ordinal data, the Mann–Whitney U test was applied. In case of multiple relevant predictors, binary outcomes were evaluated by a logistic regression model including MS-specialization and number of patients as predictors.

## Results

### Participating Practices

From 600 mailed surveys, 74 completed and returned surveys to the Multiple Sclerosis Center Dresden (12.3%). About two-thirds of the returned surveys came from neurologists with practices treating neurological and psychiatric disorders (48/74, 65%) whereas one-third were returned from purely neurological practices (26/74, 35%). As much as 32 practices (43%) featured a specialization in MS and 17 (23%) reported additional specializations like psychotherapy or epileptology. When looking at the number of patients per quarter, 32 practices (43%) stated that less than 100 patients had been treated whereas 24 (32%) treated between 100 and 200 patients and 18 (24%) medicated more than 200 patients. Practices that specialized in MS showed higher numbers of patients per quarter (median: 100-200) than other participating neurological practices (median: <100, *P*<.001).

### Health Information Technology Infrastructure of Neurological Practices

Of the 10 practices, 9 were already connected to the Internet (67/74) but only 49% (36/74) preferred a permanent access. The Internet has been utilized by 82% (67/74) as a source for research, by 46% (34/74) for medical studies, by 31% (23/74) for noninterventional studies, by 19% (14/74) for clinical documentation, by 5% (4/74) for accounting, and by 4% (3/74) for email communication with patients.

Almost every practice (73/74) possessed at least one computer for documentation purposes. The most common type of HIT infrastructure was a complete practice network with several access points (65/74, 88%). The ability to access patient data network-wide was preferred (49/74, 66%). Nonetheless, some health care professionals chose documentation limited to a single device (24/74, 32%). Considering data sharing with research registers, 43% (31/73) opted for an online interface whereas 58% (42/73) decided on offline data transmission. Practices that specialized in MS in comparison with other neurological practices presented an increased interest in online documentation (Table 1). Different numbers of patients per quarter did not result in statistically significant different answers.

**Table 1:** Information technology infrastructure of neurological practices.

		Practices specialized in MS, n (%)	Other neurological practices, n (%)	*P* value
Existing Internet access		31/32 (97)	36/42 (86)	.13^a^
Continuous Internet connection		20/32 (63)	16/42 (38)	.04^b^
**Internet is used for...**				
	Research	27/32 (84)	34/42 (81)	.70^b^
	Documentation of interventional studies	22/32 (69)	28.6% (12/42)	<.001^b^
	Documentation of noninterventional studies	15/32 (47)	8/42 (19)	.010^b^
	Clinical documentation	11/32 (34)	3/42 (7)	.003^b^
**Documentation via...** ^c^				.44^b^
	Network	23/32 (72)	26/41 (63)	
	Single device	9/32 (28)	15/41 (37)	
**Preferred method of data transmission** ^c^				.10^b^
	Online	17/32 (53)	14/41 (34)	
	Offline	15/32 (47)	27/41 (66)	

^a^Fisher’s exact test

^b^Chi-square test

^c^Reduced sample size due to missing values

### eHealth Services for Daily Care

The majority of participating practices considered eHealth services as definitely useful (doctor: 18/74, 24%; nurse: 19/74, 26%) or at least partially useful (doctor: 52/74, 70%; nurse: 49/74, 66%) for doctor’s business and nurse duties whereas only a small minority doubted their usefulness (doctor: 4/74, 5%; nurse: 6/74, 8%). The highest potential for benefits of HIT were seen in clinical documentation (61/74, 82%), followed by protection against recourse (47/74, 64%), documentation of medical studies (42/74, 57%), and documentation of noninterventional studies (38/74, 51%). In this regard, physicians’ assumed benefits did not differ from those of other practice staff members. When looking at specific tasks, retrieving patient data relevant for the treatment process (yes: 34/72, 47%; partially: 35/72, 49%; no: 3/72, 4%) and diagnosing specific MS symptoms and courses of disease (yes: 33/74, 45%; partially: 34/74, 46%; no: 7/74, 10%) received the highest ratings for being potentially improved by HIT. Beyond that, HIT may enhance physician-patient communication (yes: 25/73, 34%; partially: 38/73, 52%; no: 10/73, 14%) and patient education (yes: 20/72, 28%; partially: 36/72, 50%; no: 16/72, 22%). An increased precision in the assessment of MS-specific scales (1/74) and the support of practice management in general (3/74) were mentioned as additional benefits. The appreciation of eHealth services tended to be higher in practices specialized in MS than those in other neurological practices (Table 2).

**Table 2:** Usefulness of health information technology in neurological practices.

		Practices specialized in MS, n (%)	Other neurological practices, n (%)	*P* values
**eHealth services are useful for doctors’ duties**				<.001^a^
	Yes	15/32 (47)	3/42 (7)	
	Partially	16/32 (50)	36/42 (86)	
	No	1/32 (3)	3/42 (7)	
**eHealth services are useful for nurses’ duties**				.03^a^
	Yes	15/32 (47)	4/42 (10)	
	Partially	17/32 (53)	32/42 (76)	
	No	0/32	6/42 (14)	
**eHealth services are useful for...**				
	Recourses	23/32 (72)	24/42 (57)	.19^b^
	Clinical documentation	28/32 (88)	33/42 (79)	.32^b^
	Documentation of interventional studies	27/32 (84)	15/42 (36)	<.001^b^
	Documentation of noninterventional studies	28/32 (88)	10/42 (24)	<.001^b^
**eHealth services are useful for patient education** ^c^				.16^a^
	Yes	11/32 (34)	9/40 (23)	
	Partially	16/32 (50)	20/40 (50)	
	No	5/32 (16)	11/40 (28)	
**eHealth services are useful for physician-patient communication** ^c^				.23^a^
	Yes	13/32 (41)	12/41 (29)	
	Partially	16/32 (50)	22/41 (54)	
	No	3/32 (9)	7/41 (17)	
**eHealth services are useful for retrieving patient data** ^c^				.25^a^
	Yes	17/32 (53)	17/40 (43)	
	Partially	15/32 (47)	20/40 (50)	
	No	0/32	3/40 (8)	
**eHealth services are useful for diagnosing specific MS symptoms and courses of disease**				.34^a^
	Yes	16/32 (50)	17/42 (41)	
	Partially	14/32 (44)	20/42 (48)	
	No	2/32 (6)	5/42 (12)	

^a^Mann-Whitney U test

^b^Chi-square test

^c^Reduced sample size due to missing values

### Electronic Health Records for Multiple Sclerosis

Among the participating centers, 91% (67/74) welcomed the opportunity of a specific clinical documentation for MS and 87% (64/74) showed great interest in an extended and more interconnected electronic documentation of MS patients. Clinical parameters (59/74, 80%) were most important in documentation, followed by symptomatic parameters like measures of fatigue or depression (53/74, 72%) and quality of life (47/74, 64%). Given the chance to communicate additional desirable parameters, many options were reported: from tests for cognition and working ability to results from magnetic resonance imaging and cerebrospinal fluid, medication history, social factors (eg, family status, job status), visit structures for prominent disease modifying drugs, and a broad approach to common disabilities in MS. The request for an integration into clinical networks (53/74, 72%) significantly exceeded (*P*=.005) the request for the ability to import data from other systems (35/74, 47%), which was still considerably high. Further design tasks for EHRs were specified: the ability to support communication and data exchange with general practitioners, the integration of data management tools, an easy-to-use design, verified compatibility with other systems, data security, and possibilities to avoid double documentation in several documentation systems. Neither type of neurological practice differed in opinions about EHR systems for MS.

### Recognition of the Multiple Sclerosis Documentation System

In nearly half of the participating practices (34/74, 46%), the Multiple Sclerosis Documentation System (MSDS) was already known. The level of awareness was higher among practices specialized in MS (23/32, 72%) than among other neurological practices (11/42, 26%) (*P*<.001). Fifteen practices already used one version of MSDS (MSDS Practice, Bayer Healthcare). Reasons for not using MSDS were concerns about double documentation (8/74) and the expected expenditure of time (9/74).

## Discussion

### Principal Findings

In the process of HIT development, a user-driven needs assessment ensures the inclusion of health care professional perspectives and, therefore, supports the realization of benefits of HIT. In order to examine this issue, we surveyed neurological health care professionals in Germany and their handling of HIT, especially of electronic patient management systems and EHRs, and included the results in the development process of the MSDS^3D^. Looking at the results, the adoption of HIT in daily health care was quite high among neurological practices and clinics and even higher among practices specialized in MS. In general, respondents were very open-minded about eHealth services. Highest potential benefits of HIT were seen in treatment documentation and study documentation. When designing interfaces of complex eHealth services for neurological practices and clinics, options for online transmission as well as for offline transfer should be implemented, and the ability to connect with preexisting HIT structures should be assured. An MS-specific EHR system would be welcomed by the majority of participating practices.

### eHealth Services for Neurological Practices

Health information technology may improve quality of care by increasing adherence to guidelines and decreasing medication errors [2]. Before this study, data on the use and acceptance of HIT by neurologists and chronic care providers in the domain of MS was lacking. Our study showed that there is a high base rate of IT adoption among neurological practices and that practices specialized in MS present an increased interest in documentation and patient management assisted by eHealth services. These results supported the assumption that the domain of MS is a promising field for upcoming eHealth trends. In addition, rates of HIT adoption did not differ by practice size measured as number of patients per quarter.

We found that eHealth services were perceived as generally useful for physicians and nurses in neurological practices with highest capabilities for improvements in clinical documentation, data acquisition, diagnosis of specific MS symptoms, physician-patient communication, and patient education. Practices specialized in MS had an increased need for eHealth services for documentation purposes of interventional and noninterventional studies. The most prominent reason for nonadoption of eHealth services was the concern about additional expenditure of time for documentation. The results were in line with other works on the benefits of HIT adoption. Mickan et al proposed four functional aspects that may be improved by mobile eHealth services: patient documentation, patient care, health information seeking, and professional work patterns [59]. Clinical pathways as representatives of such work patterns were associated with reduced in-hospital complications and improved documentation [60]. Nonetheless, mixed results were available about whether eHealth services may lead to a reduction or an increase in the time required for documentation [48,60,61].

The integration of patient data into larger systems of health data management remains an essential task to fulfill [1]. According to the responses in our survey, emphasis has to be laid on a dual-option for data transmission (online and offline mode) and on an extensive integration of standard interfaces for common research and health care networks during the development of a local EHR system.

### Electronic Health Records for Multiple Sclerosis

There is a growing trend for adoption of EHRs within the past decade. Some authors reported a yearly increase of 10% [5,21,51]. In 2013, about 70% of US physicians had already implemented at least a basic version of an EHR whereas only 9% declared themselves as “persistent nonadopters” [51]. Those nonadopters were characterized as mostly elder physicians with rarely more than 2 physicians per practice. In our survey, a similar rate of physicians doubting the usefulness of HIT was found. But looking at the rate of adoption among practices specialized in MS, the rate of nonadopters tended toward zero. The vast majority of the responding practices welcomed the opportunity of electronically assisted clinical documentation for MS. Clinical parameters and scores like the Expanded Disability Status Scale were highly appreciated for integration into an EHR for MS, followed by symptomatic parameters like measures of fatigue or depression and patient-reported outcomes like measures of health-related quality of life. Additionally, the import of data from preexisting databases and the integration into clinical networks must be secured in order to meet neurologists’ needs. Likewise, Kruse et al indicated that the adoption of an EHR or a computerized physician order entry were predominantly associated with internal organizational factors that must be taken into account [21].

MSDS is the most widely used electronic documentation system for patients with MS in Germany [35]. In an evidence-based and user-driven development process, MSDS has evolved from a database to a complete patient management system [26,42,44,46,52]. In about half of the participating neurological practices, MSDS was already known, especially among practices specialized in MS (72%). Furthermore, 20% of all responders already used a version of MSDS. Results and lessons of the current survey have been integrated into the continued development of MSDS^3D^, the current version of MSDS, which can be used by patients, nurses, and physicians to enhance data collection and facilitate an interactive analysis and interpretation of given results via touch screen devices or other devices via the Internet (by app, email, or web browser) in neurological practices.

### Limitations

Only an average response rate of about 12% was achieved in this postal survey, which may have limited the variety of reported additional aspects of important EHR features and the representativeness of the given results. Despite that, no type of neurological practice (with respect to specialization and patient numbers) was underrepresented among the responding practices, and responders were clearly not restricted to the portion of practices being familiar with the system MSDS. A detailed characterization of nonresponders was not within the scope of this study. Some factors associated with the adoption of eHealth services in other studies like physician’s age or the number of staff members were not included in the questionnaire. Moreover, data on the use of mobile devices could have improved the illustration of HIT usage.

### Conclusions

In this study, we surveyed the use of HIT in neurological practices in Germany and the perceived usefulness of eHealth services like EHRs for the community of MS health care professionals. Both physicians and nurses may significantly benefit from electronically assisted documentation and patient management. Many aspects of patient documentation and education will be enhanced by eHealth services if the most informative measures are integrated in an easy-to-use and easily connectable approach. MS-specific eHealth services were highly appreciated, but the current level of adoption is still behind the level of interest in an extended and more interconnected electronic documentation of MS patients. A comprehensive electronic patient management system should incorporate the balanced interests and needs of all agents (physician, staff members, patients, and researchers) in the field of chronic disease management. Further research should validate the presented results and increase the knowledge about the adoption of different types of HIT and applicable devices. A comparison of the electronically assisted management of different chronic diseases and the support of a multilanguage user interface may extend the application range of existing eHealth technologies and thereby raise the cost-effectiveness of such systems.

## Multimedia Appendix 1

Questionnaire on the use of health information technology in neurological practices and on the needs of neurologists for future eHealth services.

## Abbreviations

EHR: electronic health records
HIT: health information technology
MS: multiple sclerosis
MSDS: Multiple Sclerosis Documentation System
MSDS3D: Multiple Sclerosis Documentation System 3D
