# Supplementary material for: Designing an Electronic Patient Management System for Multiple Sclerosis: Building a Next Generation Multiple Sclerosis Documentation System
Source: Interact J Med Res. 2016 Jan 8;5(1):e2. doi: 10.2196/ijmr.4549 (PMC4723723; doi:10.2196/ijmr.4549)
Supplement: Supplementary file 1 [file ijmr_v5i1e2_app1.pdf]

Multimedia Appendix 1. Questionnaire on the use of health information technology in neurological practices and on the needs of neurologists for future eHealth services.

No.	*Question/Answers*
**[1]**	**You are working as ...**
[1a]	Neurologist & Psychiatrist
[1b]	Neurologist
[1c]	Further qualifications: (Free text)
**[2]**	**Do you work in a practice which is specialized in MS?**
[2a]	Yes
[2b]	No
**[3]**	**How many patients have been treated in your practice within the last quarter?**
[3a]	Less than 100
[3b]	Between 100 and 200
[3c]	More than 200
**[4]**	**You are working in ...**
[4a]	a private practice
[4b]	a hospital
**[5]**	**Do you access the Internet in your practice?**
[5a]	Yes
[5b]	No
**[5c]**	**If so, what do you use it for? (Multiple answers possible)**
[5c1]	Research (e.g. addresses, latest study results)
[5c2]	Clinical documentation
[5c3]	Documentation on intervention studies
[5c4]	Documentation on non-intervention studies (NIS)
[5c5]	Other: (Free text)
**[6]**	**How is patient data documented?**
[6a]	Documentation of patient data via an IT network
[6b]	Documentation of patient data on a single IT device
[6c]	Other: (Free text)
**[7]**	**Are the IT devices in your practice continuously connected to the Internet?**
[7a]	Yes
[7b]	No
**[8]**	**Which type of data transmission do you prefer (e.g. for connection with**

No.	*Question/Answers*
	**research registers)?**
[8a]	Online (via continuous Internet connection)
[8b]	Offline
**[9]**	**How can eHealth services assist <u>you</u> in your daily duties as a physician?**
[9a]	eHealth services can be beneficial in any way.
[9b]	eHealth services do not provide any use to me.
[9c]	eHealth services can partially be beneficial to me.
**[9d]**	**If so, in which aspects can eHealth services assist <u>you</u> in your daily duties as a physician? (Multiple answers possible)**
[9d1]	*Protection* against recourses and for audits
[9d2]	Clinical documentation
[9d3]	Documentation on intervention studies
[9d4]	Documentation on non-intervention studies (NIS)
[9d5]	Other: (Free text)
**[10]**	**How can eHealth services assist <u>your nurses</u> in their daily duties?**
[10a]	eHealth services can be beneficial in any way.
[10b]	eHealth services do not provide any use to them.
[10c]	eHealth services can partially be beneficial to them.
**[10d]**	**If so, in which aspects can eHealth services assist <u>your nurses</u> in their daily duties? (Multiple answers possible)**
[10d1]	Clinical documentation
[10d2]	Documentation on intervention studies
[10d3]	Documentation on non-intervention studies (NIS)
[10d4]	Other: (Free text)
**[11]**	**Would you like to share information with your patients for patient education via eHealth services?**
[11a]	Yes
[11b]	No
[11c]	Partially yes

No.	*Question/Answers*

**[12]**	**Can eHealth services assist you in your physician-patient-communication?**
[12a]	Yes
[12b]	No
[12c]	Partially yes
**[13]**	**Can eHealth services assist you in retrieving patient data?**
[13a]	Yes
[13b]	No
[13c]	Partially yes
**[14]**	**Can eHealth services assist you in diagnosing specific MS symptoms and courses of disease?**
[14a]	Yes
[14b]	No
[14c]	Partially yes
**[15]**	**Do you consider an MS-specific clinical documentation desirable?**
[15a]	Yes
[15b]	No
**[15c]**	**If so, which aspects should be considered? (Multiple answers possible)**
[15c1]	Clinical parameters (e.g. relapse rate, EDSS, progression)
[15c2]	Parameters of quality of life
[15c3]	Symptomatic parameters (e.g. for depression, fatigue)
[15c4]	Further clinical parameters: (Free text)
[15c5]	Further patient-related parameters: (Free text)
[15c6]	Other parameters: (Free text)
**[16]**	**Do you consider a more connected electronic MS-specific clinical documentation desirable?**
[16a]	Yes
[16b]	No

No.	*Question/Answers*
**[17]**	**Future electronic documentation systems should assist you in …** **(Multiple answers possible)**
[17a]	Import of data from other systems
[17b]	Integration into clinical or research networks
[17c]	Further aspects: (Free text)
**[18]**	**Do you know the Multiple Sclerosis Documentation System (MSDS)?**
[18a]	Yes
[18b]	No
**[18c]**	**If so, which version do you use?**
[18c1]	MSDS Praxis
[18c2]	MSDS Clinic
[18c3]	None
**[18d]**	**Are there any reasons for not using MSDS? (Free text)**
